# The Molecular Mechanisms by Which Vitamin D Prevents Insulin Resistance and Associated Disorders

**DOI:** 10.3390/ijms21186644

**Published:** 2020-09-11

**Authors:** Izabela Szymczak-Pajor, Józef Drzewoski, Agnieszka Śliwińska

**Affiliations:** 1 Department of Nucleic Acid Biochemistry, Medical University of Lodz, 251 Pomorska Str., 92-213 Lodz, Poland; izabela.szymczak@umed.lodz.pl; 2 Central Teaching Hospital of the Medical University of Lodz, 251 Pomorska Str., 92-213 Lodz, Poland; jozef.drzewoski@umed.lodz.pl

**Keywords:** vitamin D deficiency, insulin resistance, adipose tissue, oxidative stress, sub-inflammation, epigenetic modification

## Abstract

Numerous studies have shown that vitamin D deficiency is very common in modern societies and is perceived as an important risk factor in the development of insulin resistance and related diseases such as obesity and type 2 diabetes (T2DM). While it is generally accepted that vitamin D is a regulator of bone homeostasis, its ability to counteract insulin resistance is subject to debate. The goal of this communication is to review the molecular mechanism by which vitamin D reduces insulin resistance and related complications. The university library, PUBMED, and Google Scholar were searched to find relevant studies to be summarized in this review article. Insulin resistance is accompanied by chronic hyperglycaemia and inflammation. Recent studies have shown that vitamin D exhibits indirect antioxidative properties and participates in the maintenance of normal resting ROS level. Appealingly, vitamin D reduces inflammation and regulates Ca^2+^ level in many cell types. Therefore, the beneficial actions of vitamin D include diminished insulin resistance which is observed as an improvement of glucose and lipid metabolism in insulin-sensitive tissues.

## 1. Introduction

There is mounting evidence that vitamin D deficiency is now a worldwide health problem. In addition, an alarming number of diseases connected with vitamin D deficiency such as obesity and type 2 diabetes mellitus (T2DM) are observed. Both basic and clinical studies demonstrated that the majority of common characteristics of these diseases result from defects in insulin signaling, systemic inflammation, and pancreatic β-cells dysfunction [1,2,3,4]. It should be stressed that according to recent investigations one of the major causative factors in insulin resistance development is vitamin D deficiency. The results of some clinical studies have demonstrated that vitamin D supplementation improves major metabolic parameters associated with insulin resistance, including low-density lipoprotein (LDL), total cholesterol (TC), glycated hemoglobin (HbA1c), triglyceride (TAG), and homeostatic model assessment-insulin resistance (HOMA-IR). We have shown that three-month supplementation with vitamin D of the elderly with metabolic disorders markedly elevates HDL level, reduces HOMA-IR, and TG/HDL ratio. Moreover, we observed that HbA1c percentage decreased about 0.5% in T2DM patients after vitamin D supplementation [5]. In turn, Upreti et al. have revealed that six-month supplementation with vitamin D of T2DM patients leads to distinct reduction of HbA1c [6]. The results of study carried out by Mirrhosseini et al. have showed that vitamin D decreases HbA1c, fasting plasma glucose (FPG), and HOMA-IR contributing to better glycemic control [7]. Interestingly, Tabesh et al. have observed that co-supplementation of vitamin D with calcium decreases serum insulin level, HbA1c, HOMA-IR, LDL, and TC/HDL. Additionally, they also detected the significant elevation of quantitative insulin sensitivity check index (QUICKI) and HDL [8]. El Hajj et al. have found that vitamin D triggers to significantly diminish of HOMA-IR, FPG, TC, and LDL, but without any significant changes in HbA1c [9]. The results of studies conducted by Barzegardi et al. have presented pronounced decrease in serum levels of TG, LDL, and TC in diabetic nephropathy patients after supplementation with vitamin D [10]. Taken together, these observations support that vitamin D improves metabolic control of diabetes.

Vitamin D is involved in many cellular processes, e.g., the presence of its receptor and its metabolizing enzymes have been found in the cells of various tissues, including pancreatic β-cells, adipocytes, hepatocytes, and myocytes [11,12,13,14]. It also controls blood glucose concentration by regulating insulin secretion and insulin sensitivity [15]. Furthermore, it has been found to act in adipose tissue which is a major storage site of the vitamin [11]. It should be underlined that adipose tissue secretes numerous adipocytokines involved in inflammation, a typical feature of insulin resistance, obesity, and T2DM [11]. Numerous studies have revealed that vitamin D reduces the extent of inflammation and chronic hyperglycemia-generated oxidative stress [5,15]. Appealingly, vitamin D was demonstrated to modulate hepatic lipid and glucose metabolism [16]. Finally, it has also been shown that vitamin D counteracts diet-induced insulin resistance in skeletal muscle [17].

However, it should be also emphasized that the results of clinical studies have revealed no effect of vitamin D on insulin resistance and related disorders, including oxidative stress and inflammation. Lerchbaum et al. have shown that vitamin D supplementation did not change significantly metabolic parameters regarding insulin resistance and lipids in heathy men [18]. Forouhi et al. have found no effect of vitamin D on HbA1c, lipid and apolipoprotein levels, CRP, as well as anthropometric measures in subjects with increased risk of T2DM [19]. Similarly, Heshmat et al. have revealed no changes in HbA1c, anthropometric measures, and HOMA-IR in diabetic patients treated with vitamin D [20]. No differences in the FPG oral glucose tolerance test (OGTT) between prediabetes subjects supplemented with vitamin D in comparison to the placebo group have also been observed [21]. In addition, no significant changes between T2DM group and T2DM group supplemented with vitamin D have also been observed in the hs-CRP level, oxidative stress markers, LDL, HDL, and HbA1c [22]. In turn, Asemi et al. did not observe any significant changes in total plasma glutathione (GSH) and serum high sensitivity C-reactive protein (hs-CRP) level in pregnant women with gestational diabetes after supplementation with vitamin D [23].

Considering the above, the aim of this review is to provide a molecular insight into how vitamin D reduces insulin resistance and its consequences.

## 2. Methods

To summarize the current scientific literature devoted to the molecular mechanism involved in the reduction of insulin resistance and its consequences brought about by vitamin D, the university library, PUBMED, and Google Scholar were searched to identify the relevant articles. The following keyword combinations were used: Vitamin D OR vitamin D action OR vitamin D receptor OR genomic action of vitamin D OR non-genomic action of vitamin D OR molecular mechanism of vitamin D OR vitamin D deficiency OR vitamin D insufficiency OR vitamin D supplementation AND insulin resistance OR intracellular calcium level OR insulin secretion OR insulin sensitivity OR insulin signaling OR pancreatic β-cells dysfunction OR skeletal muscle OR myocytes OR liver OR hepatocytes OR adipose tissue OR adipocytes OR adipogenesis OR adipocyte apoptosis OR adipocytokines OR adiponectin OR lipid metabolism OR glucose metabolism OR thermogenesis OR sub-inflammation OR epigenetic control OR oxidative stress OR reactive oxygen species OR immunomodulation OR immune cells. Subject to analysis were in vivo, in vitro, animal, as well as human studies, including clinical trials.

## 3. Vitamin D in Brief

Vitamin D, a cholesterol derivative, is one of the fat soluble vitamins. The term vitamin D refers to two forms: Ergocalciferol (D_2_) and cholecalciferol (D_3_) [24,25]. Vitamin D_2_ is formed by plants and mushrooms under ultraviolet B (UVB) radiation. Vitamin D_3_, on the other hand, is synthesized in the epidermis, where pro-vitamin D_3_-7-dehydrocholesterol, is transformed into pre-vitamin D_3_ under 290–315 nm UVB radiation. Subsequently, pre-vitamin D_3_ is converted into vitamin D_3_ in a heat-dependent process. It should be pointed out that about 20% of vitamin D comes from our diet, the remaining 80% being provided by our skin. Both vitamin D_2_ and D_3_ bind to the vitamin D-binding protein (VDBP) in the blood and are transported to the liver, where vitamin D 25-hydroxylase (CYP27A1 and CYP2R1) metabolizes it to 25-hydroxyvitamin D (25(OH)D) called calcidiol. The latter is a major circulating vitamin D form in the serum [26,27,28]. Calcidiol is further metabolized to 1,25-dihydroxyvitamin D (1,25(OH)_2_D_3_; calcitriol) by 25(OH)D 1α-hydroxylase (CYP27B1) in the proximal tubule of the kidney. Notably, CYP27B1 is expressed not only in the tubule of the kidney, but also in other cell types, including adipocytes, pancreatic β-cells, and macrophages. In turn, calcitriol is the most bioactive form of vitamin D that enters the circulation, binds to VDBP, and is then delivered to the target tissues, i.e., bone, kidney, and gut. It should be stressed that calcitriol has structural similarities to other steroid hormones, and for that reason it is classified as a hormone [24,26,27,29,30,31]. The level of calcidiol and calcitriol is regulated by 25(OH)D 24-hydroxylase (CYP24A1). The latter is a key vitamin D inactivating enzyme that performs hydroxylation of C-23 and C-24 of calcitriol and calcidiol. The inactivation of vitamin D occurs via two pathways, biliary excreted calcitroic acid is a product of the 24-hydroxylase pathway, whereas 1,25–26,23 lactone is formed in the 23-hydroxylase pathway [26,27].

In the cells of target tissues, calcitriol binds to the vitamin D receptor (VDR), which belongs to the nuclear receptor family and acts as a ligand-activated transcription factor, inducing both genomic and non-genomic response to vitamin D [32,33]. In the genomic pathway, 1,25(OH)_2_D_3_ interacts with cytosolic VDR, which connects with retinoid X receptor (RXR). The formed complex translocates to the nucleus. Subsequently, the 1,25(OH)_2_D_3_–VDR–RXR complex links with the vitamin D response element (VDRE) in the promoter region of vitamin D-responsive genes leading to recruitment of various enzymatic co-regulatory complexes engaged in facilitating the histones’ epigenetic modification, chromatin remodeling, and the recruitment of local RNA polymerase II. In consequence, the expression of numerous vitamin D-responsive genes is regulated. Vitamin D responsive genes govern multiple processes such as differentiation, proliferation, angiogenesis, metabolism, and immunomodulation [34,35,36].

In turn, the activation of the non-genomic pathway by 1,25(OH)_2_D_3_ involves its binding with membrane VDR known as 1,25D-membrane-associated, a rapid response steroid-binding protein (1,25D-MARRS). The interplay between 1,25(OH)_2_D_3_ and 1,25D-MARRS switches on multiple cell signaling pathways via direct protein-protein interaction with numerous intracellular molecules [33,37]. The non-genomic pathway activated by vitamin D turns on numerous signaling molecules, including mitogen-activated protein kinases (MAPK)s, phosphatidylinositol-3 kinase (PI3K), Ca^2+^-calmodulin kinase II (CaMPKII), phospholipase C (PLC), protein kinase A (PKA), protein kinase C (PKC), and src. The plethora of kinases activated by vitamin D transduct the signal to the following transcription factors: RXR, SP1, and SP3, which subsequently bind to VDRE on the promoter of vitamin D-responsive genes. The activation of non-genomic pathway is a rapid response to 1,25(OH)_2_D_3_ based on numerous protein-protein interactions. Simultaneously, vitamin D is also engaged in the secretion of second messengers, such as Ca^2+^, cyclic AMP, 3-phosphoinositides, and fatty acids. However, it should be underlined that the type of signaling molecules activated by vitamin D depends on the cell type and the status of its maturation [38].

**Figure 1:**
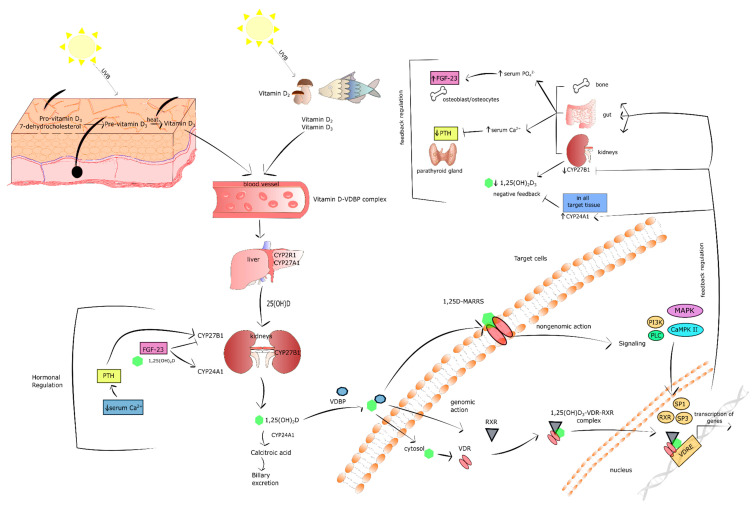
The overview of vitamin D. Stimulatory interactions are expressed by arrows and inhibition by T-bars. ↓ denotes decrease ↑ denotes increase.

Vitamin D metabolism is regulated by the level of 1,25(OH)_2_D_3_ in a negative feedback mechanism [39,40]. Vitamin D inactivating enzyme CYP24A1 is a transcriptional target of 1,25(OH)_2_D_3_–VDR–RXR complex. The promoter region of CYP24A1 includes two VDREs that are responsible for the induction of CYP24A1 by 1,25(OH)_2_D_3_ [41]. Moreover, 1,25(OH)_2_D_3_ stimulates the expression of CYP24A1 by the recruitment of RNA II polymerase and histone H4 acetyltransferases to CYP24A1 gene [42]. Therefore, the level of both calcidiol and calcitriol is regulated by 1,25(OH)_2_D_3_-mediated CYP24A1 expression in the kidney. Furthermore, 1,25(OH)_2_D_3_ suppresses CYP27B1 transcription in the kidney by complex mechanisms engaging epigenetic modifications of CYP27B1 promoter region [43]. Additionally, studies revealed that vitamin D metabolism is regulated by fibroblast growth factor-23 (FGF-23) and parathyroid hormone (PTH). These biomolecules play a key role in the maintenance of Ca^2+^ and phosphate homeostasis [44,45,46]. FGF-23 is a hormone secreted by osteocytes and osteoblasts in response to both high serum level of 1,25(OH)_2_D_3_ and phosphate [45]. On the one hand, FGF-23 facilitates the secretion of phosphate by suppressing the expression of sodium-phosphate cotransporter 2 (NPT2) placed at the apical membranes of proximal renal tubules. On the other hand, FGF-23 decreases serum levels of 1,25(OH)_2_D_3_ by downregulation of CYP27B1 and upregulation of CYP24A1 in the kidney [47,48,49]. In turn, the parathyroid gland secretes PTH in a response to low level of Ca^2+^ in the serum [44]. PTH induces renal expression of CYP27B1 causing an increase of 1,25(OH)_2_D_3_ production [50,51]. It should be recognized that increased 1,25(OH)_2_D_3_ stimulates its own degradation via the activation of CYP24A1 expression, whereas PTH sustains the 1,25(OH)_2_D_3_ level by the kidney induction of CYP24A1 mRNA degradation [52,53]. Notably, a high Ca^2+^ level resulting in prolonged induction of 1,25(OH)_2_D_3_ negatively regulates PTH secretion by the parathyroid gland in a mechanism of negative feedback mechanism [54]. Figure 1 presents key information about vitamin D as described above.

### Vitamin D Level

The serum level of calcidiol (25(OH)D) is thought to be a vitamin D status marker that reflects the actual amount of vitamin D in an organism [55,56]. A deficiency in vitamin D is considered primarily 25(OH)D concentration below 25–30 nmol/L (10–12 ng/mL). There is no common agreement as to the optimal concentration of vitamin D, however it is generally accepted that the serum 25(OH)D level should not be lower than 50 nmol/L by the Scandinavian Nutrition Societies, The North American Institute of Medicine (IOM), the European Society for Clinical and Economic Aspects of Osteoporosis and Osteoarthritis, the D-A-CH nutrition societies, and the German Osteology governing body (DVO) [57,58]. On the other hand, the International Osteoporosis Foundation and the Endocrine Society considers 75 nmol/L (30 ng/mL) of the calcidiol in the serum as an adequate vitamin D level.

It should be also underlined that the excess of vitamin D is toxic and manifests itself as a severe hypercalcemia [59]. Carmo et al. have demonstrated extensive vascular remodeling and elevated vascular calcification as a response to high doses of vitamin D in a murine model of obesity and insulin resistance [60]. It should be emphasized that vitamin D presents biphasic dose-responses (hormesis). In low doses it shows beneficial effect, but in high doses vitamin D is a toxic agent.

The percentage of people with vitamin D deficiency is continuously rising, especially in countries with low sun exposure. The main causes of vitamin D deficiency are alterations in vitamin D transformation and metabolism such as impaired absorption, increased catabolism, elevated urinary loss of 25(OH)D, reduced synthesis, and impaired transport. Vitamin D deficiency may also be due to geographical factors, lifestyle, individual variables (i.e., skin pigmentation, skin grafts and aging), some disorders, and therapy with some drugs. Latitude, seasons, and the time of day are the geographical factors significantly affecting the volume of UVB photons that reach the earth according to the zenith angle of the sun. Latitude higher than 35°, the season from November to March, morning and late afternoon, as well as low position of the sun on the horizon are related to lower UVB index and decreased production of vitamin D in the skin [61]. The usage of sunscreens/sunoils, type of diet, obesity, breast-feeding, and indoor workplace also significantly decrease the level of vitamin D in the human body. Adipose tissue has been shown to sequestrate vitamin D, thus obesity is associated with its reduced availability [61]. It has also been noted that human milk contains a low level of vitamin D making infants susceptible to vitamin D deficiency during sole breast feeding [62,63].

Individual features such as skin pigmentation, age and skin grafts also affect the synthesis of vitamin D. Skin pigmentation depends on the concentration of melanin, which is responsible for the absorption of UVB radiation. Thus, with dark-skinned people vitamin D production is five times lower as compared to white-skinned people (Caucasian). Interestingly, skin grafts due to burns and aging reduce the level of 7-dehydrocholesterol in the skin by about 75% in people aged 70 years and older [61].

Low vitamin D level is observed in people treated with cholesterol lowering medications, undergoing bypass surgery, suffering from celiac disease and chronic inflammatory bowel disease, and other conditions [64,65]. In subjects with chronic kidney disease, on the one hand, the synthesis of 1,25(OH)_2_D_3_ is inhibited, and-on the other-its metabolites are extracted from the body in higher amounts than in individuals with normal kidney function [61]. In turn, nephrotic syndrome is associated with increased loss of 25(OH)D with urine.

A significant catabolic effect of glucocorticoid, anticonvulsants, antirejection, AIDS treatment medications, and nucleoside/nucleotide reverse transcriptase inhibitors on vitamin D metabolism was also revealed [61]. In addition, rimfapicin and carbamazepine increase the level of PTH, which decreases the active form of vitamin D and in consequence increases its clearance [66,67,68,69].

Low concentration of vitamin D was reported in carriers of polymorphisms and mutations of VDBP and CYP27B1 genes. Numerous polymorphisms and mutations in genes associated with vitamin D transport and transformation were detected in all types of rickets [61].

## 4. How does Insulin Resistance Develop?

### 4.1. The Physiology of Insulin Signaling

One of the major regulators of energy homeostasis is insulin signaling [70]. Insulin receptor (IR), a member of the tyrosine kinase family receptors, is composed of extracellular α subunit and transmembrane β subunit. IR activation occurs after insulin binding to α chain of IR that triggers structural changes in β chain. The result of IR activation is the formation of the heterotetrameric structure followed by autophosphorylation of numerous tyrosine residues that are potential docking sites for the multiple components of various signaling pathways [71]. Thus, the recruitment and phosphorylation of different adaptor proteins, including substrate proteins, i.e., insulin-receptor substrate (IRS), is mediated by multiple phosphotyrosines [72]. Next, phosphorylated IRSs activate and translocate PI3K to the plasma membrane which phosphorylates the phosphatidylinositol 4,5-bisphosphate (PIP2) to phosphatidylinositol-3,4,5-trisphosphate (PIP3). The level of PIP3 is controlled by phosphatase and tensin homolog (PTEN) and SH2-containing inositol 5’-phosphatase-2 (SHIP2) that perform dephosphorylation of PIP3 [73]. Insulin-initiated elevated level of PIP3 activates serine-threonine kinase phosphoinositide-dependent protein kinase-1 (PDK1) causing both phosphorylation and activation of PKC ζ/λ and protein kinase B also called Akt. The two proteins increase glucose uptake by the translocation of GLUT to the cell membrane, including adipocytes’ and muscle cells’ membranes [74,75,76]. Moreover, Akt stimulates glycogenesis in the muscles and liver and lipogenesis in the adipocyte tissue, as well as the synthesis of protein, but represses proteolysis, lipolysis, gluconeogenesis, and glycogenolysis [77]. Interestingly, Liang et al. reported that sirtuin 1 (SIRT1) controlled the insulin-mediated phosphorylation of IR and IRS. SIRT1 is a NAD-dependent deacetylase that positively regulates insulin signaling via deacetylation of IRS-2, phosphorylation of IRS-1, repression of protein tyrosine phosphate non-receptor type 1 (Ptpn1) expression, and the activation of Akt in insulin-responsive cells [78]. It should also be pointed out that insulin, apart from its metabolic effects, is a growth factor engaged in cell growth, proliferation, and differentiation [79]. Its mitogenic activity occurs via the induction of MAPK cascade [77].

### 4.2. The Mechanism of Insulin Resistance

The typical signs of insulin resistance state are reduced uptake of glucose by skeletal muscle, liver, and adipose tissue, and diminished gluconeogenesis in the liver [70,79]. As a result, the blood glucose level pronouncedly increases and-if prolonged-it exerts a toxic effect on all cells, including those in insulin-sensitive tissues. For example, impaired insulin response in adipocytes contributes to increased release of free fatty acids (FFAs) into the circulation where they are uptaken by various organs, mainly by the liver. In turn, chronic hyperglycaemia leads to the overproduction of reactive oxygen species (ROS) and formation of oxidative stress. It is well documented that both glucotoxicity and lipotoxicity induce chronic inflammation and each of these pathologies accelerates the development of insulin resistance [80]. Several molecular pathways have been identified to play a key role in insulin resistance [70]. FFAs and related metabolites including ceramides, diacyloglycerol (DAG), acyl-CoA act on many protein kinases, including PKC ζ/λ, PKC-θ, nuclear factor-κB (NF-κB), kinase-β [IκB kinase-β (IKK-β)], Jun kinase (JNK), and trigger to IRS phosphorylation which, in turn, attenuates insulin signaling [80,81,82,83,84].

#### 4.2.1. Muscle

**Figure 2:**
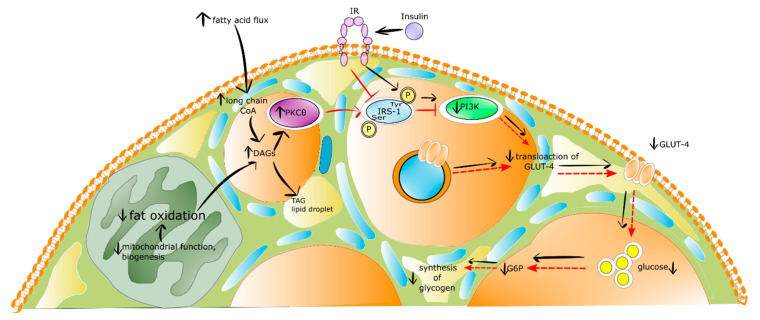
The insulin resistance mechanism in skeletal muscle. Stimulatory interactions are expressed by arrows and inhibition by T-bars, red color of arrow and T-bars denotes insulin resistance state, whereas black physiological state. ↓ denotes decrease ↑ denotes increase.

It is well recognized that the systemic, increased availability of lipids, mainly elevated flux of fatty acids, enlarges the intramyocellular pool of long-chain fatty acyl-(CoA). This results in higher energy supply to mitochondrial oxidation and the synthesis of diacylglycerols (DAGs) for storage in the form of lipid droplets filled with TAG. The intramyocellular DAG level temporarily or permanently increases when the delivery and uptake of fatty acids exceed the ratio of mitochondrial long-chain fatty acyl-CoA oxidation and DAGs to TAGs incorporation. The latter phenomena provide elevated C_18_-containing DAGs in the cytosol and membrane activated novel protein kinase C (nPKC) isoforms, i.e., PKCθ. The translocation of PKCθ into the membrane enhances serine (1101 position) phosphorylation of IRS-1, which results in the inhibition of insulin-mediated tyrosine phosphorylation of IRS-1 and downstream kinases, including PI3K. The direct consequence of these events is the restraint of the recruitment of GLUT-4 to the cell membrane, impaired phosphorylation of glucose-6-phosphate (G-6-P), and reduced synthesis of glycogen [85]. The insulin resistance in skeletal muscle is shown in Figure 2.

#### 4.2.2. Liver

**Figure 3:**
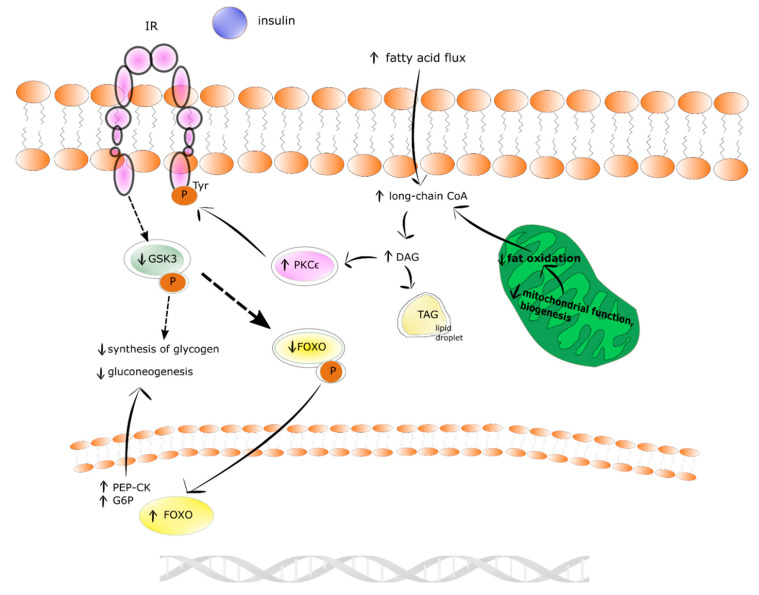
The insulin resistance mechanism in human liver. Physiological state is denoted by solid arrows and insulin resistant state by dotted arrows. ↓ denotes decrease ↑ denotes increase.

Prolonged and excessive intrahepatocellular influx of fatty acids leads to grow in the level of DAGs in the liver. In addition, the formation of DAGs in de novo lipogenesis, and re-estrification of fatty acids exceeds their incorporation into TAG in lipid droplets and mitochondrial oxidation. Subsequently, DAGs in the hepatocyte activate protein kinase C ε (PKCε) which phosphorylates tyrosines of IR. In turn, the phosphorylation of glycogen synthase kinase 3 (GSK3) and the phosphorylation of forkhead box subgroup O (FOXO) decrease. As a result of these events the activity of glycogen synthase, the storage of insulin-stimulated glycogen, and the transcription of FOXO-mediated gluconeogenic enzymes (i.e., G-6-P and phosphoenolpyruvate carboxykinase (PEP-CK)) are diminished. Finally, insulin-mediated suppression of hepatic gluconeogenesis occurs in insulin resistance-state [85]. The insulin resistance state in human liver is shown in Figure 3.

#### 4.2.3. Adipose Tissue

Similarly, to hepatocytes and myocytes, the development of insulin resistance in adipocytes is related to impaired insulin signaling. The decreased IRS1 expression and increased IRS2, that are key substrate for PI3K, were found in insulin resistance state in adipocytes [86]. Moreover, insulin resistant adipocytes present decreased expression of GLUT4 [87] and alterations in the profile of secreted adipocytokines such as leptin, tumor necrosis factor α (TNF-α), and adiponectin [88,89].

Physiologically, the level of TNF-α is very low, but it increases in obesity states, leading to the acceleration of lipolysis and inhibition of lipogenesis. Interestingly, not only adipocytes, but also cells derived from stromovascular fraction such as macrophages, endothelial cells, fibroblasts, preadipocytes, smooth muscle cells, and leukocytes are an essential source of TNF-α in adipose tissue [90,91,92,93,94]. Appealingly, it was also proposed that the TNF-α role in insulin resistance development is associated with increased serine phosphorylation of IRS1 and decreased expression of GLUT4 [95,96].

Physiologically, insulin promotes TAG accumulation in adipocytes via the stimulation of preadipocytes’ differentiation to adipocytes, elevation of glucose uptake and lipogenesis, as well as suppression of lipolysis [88,97]. The insulin action in adipocytes is mediated through two transcription factors: FOXO1 and SREBP1. SREBP1 regulates transcription of numerous adipocyte-specific genes required for fatty acid and lipid production [98,99,100]. In turn, FOXO1 that is activated by Akt, constitutes a *trans*-factor for the *cis*-element of peroxisome proliferation-activated receptor γ (PPARγ) promoters. PPARγ, being a nuclear receptor, is a key regulator of adipogenic differentiation [101], and governs the expression of numerous adipocyte-specific genes [102]. Moreover, it was demonstrated that changes in the PPARγ activity affected the synthesis of adipocytokines, i.e., leptin, adiponectin, and were associated with insulin resistance. FOXO1 binds to the PPARγ promoter and suppresses its expression. Armoni et al. showed that the impaired ability of FOXO1 to translocate into the cell nucleus led to increased activity of PPARγ [103].

## 5. How does Vitamin D Overcome Insulin Resistance and Related Disorders?

### 5.1. Vitamin D via the Regulation of Ca^2+^ Homeostasis Participates in Insulin Secretion by Pancreatic β-Cells

The secretion of insulin by the pancreatic β-cells is a consequence of elevated blood glucose concentration. Glucose molecules flow into the pancreatic β-cells via the glucose transporter 2 (GLUT-2). Then, glucose breaks down in numerous metabolic pathways, which is ultimately accompanied by ATP production. Increased ATP suppresses the ATP-sensitive K^+^ channel resulting in the depolarization of β-cell membrane followed by the activation of the L-type voltage-operated channels to produce the localized Ca^2+^ pulses crucial for the secretion of insulin [104].

Numerous studies showed that vitamin D deficiency is associated with impaired secretion of insulin by pancreatic β-cells [105,106,107,108,109]. Importantly, it was demonstrated that the supplementation with vitamin D restored proper secretion of the hormone [105,107,110]. However, it should be underlined that the findings concerning this issue are not unambiguous especially with regard to clinical trials [3,111,112,113,114,115].

One of the molecular mechanisms by which vitamin D participates in insulin secretion by pancreatic β-cells is the regulation of intracellular Ca^2+^ concentration. It was reported that 1,25(OH)_2_D_3_ reduced the expression of the L-type Ca^2+^ channels causing a decrease in intracellular Ca^2+^ concentration and thereby altering calcium signaling. In turn, rapid, non-genomic 1,25(OH)_2_D_3_ action was found to be responsible for the increase of cytoplasmic Ca^2+^ level that activates exocytosis of insulin in the pancreatic β-cells. Two vitamin D-mediated signaling pathways are involved in this process. The first includes PKA activation that phosphorylates various proteins engaged in the function of L-type voltage-dependent Ca^2+^ channels associated with insulin secretion. The second engages PLC synthesis and the activation of inositol triphosphate (InsP_3_) triggering the secretion of Ca^2+^ from ER leading to DAG synthesis. Subsequently, DAG activates PKC that is responsible for the phosphorylation of the K_ATP_ channels and L-type voltage-dependent Ca^2+^ channels. The latter trigger the depolarization of cytoplasmic membrane and opening of T-type Ca^2+^ and L-type channels that in consequence leads to the elevation of intracellular Ca^2+^ followed by insulin secretion [116]. PKC is also able to mobilize insulin secretory vesicles that together with increased Ca^2+^ concentration induce insulin secretion [117]. Furthermore, increased intracellular Ca^2+^ concentration stimulates insulin secretion via activation of CaMPKII. CaMPKII is a serine-threonine protein kinase occurring in secretory vesicles of insulin. Its primary function is the promotion of phosphorylation of numerous proteins involved in exocytosis, as well as mobilization of insulin vesicles [118]. Another study demonstrated that increased intracellular Ca^2+^ concentration is associated with the expression of insulin gene via cAMP-responsive element-binding protein (CREB). The activation of CREB occurs in response to numerous stimuli, including glucose growth factors (i.e., the insulin-like growth factor-1 (IGF-1)), incretin hormones (i.e., the glucagon-like peptide-1 (GLP-1), the gastric inhibitory polypeptide (GIP), the pituitary adenylate cyclase-activating polypeptide (PACAP)). All these stimuli lead to the phosphorylation of CREB at serine 133 residue. CREB is a crucial transcriptional element responsible for the efficient transcription of insulin gene, glucose sensing, exocytosis of insulin, and survival of pancreatic β-cells [119].

It is worth highlighting that the regulation of intracellular Ca^2+^ level by vitamin D is mediated by calbidin, a cytosolic Ca^2+^ -binding protein involved in the stimulation of insulin secretion. Calbidin-D28k expression was found to be regulated by vitamin D [113,120]. It was also reported that 1,25(OH)_2_D_3_ increased the expression of calbindin D-9k, parvalbumin, the plasma membrane Ca^2+^-ATPase 1b, the sodium/calcium exchanger (NCX), and the Ca^2+^ pumps. All of these proteins are involved in the maintenance of low resting Ca^2+^ concentration in pancreatic β-cells [32,121,122,123].

**Figure 4:**
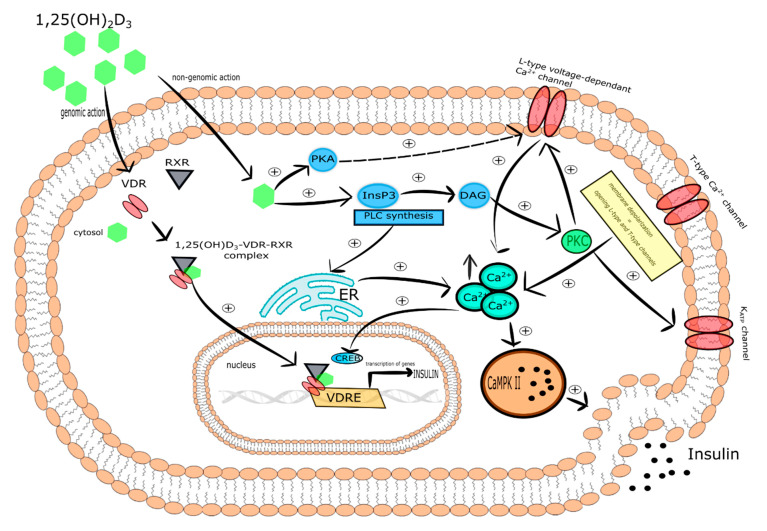
The effect of vitamin D on pancreatic β-cells. Stimulatory interactions are indicated by solid arrows and attenuation by dotted arrows. Enhancement is expressed by +. ↑ denotes increase.

Taken together, vitamin D is a potential modulator of depolarization-induced secretion of insulin via intracellular Ca^2+^ level regulation in pancreatic β-cells [120]. The effect of vitamin D on pancreatic β-cells is presented in Figure 4.

### 5.2. Vitamin D Controls Ca^2+^ Level in Myocytes and Adipocytes

It is well known that Ca^2+^ are second messengers engaged in intracellular events induced by insulin in muscle and adipose tissue. That is why intracellular Ca^2+^ level changes have a substantial impact on multidirectional insulin actions. Numerous studies have demonstrated that the low level of Ca^2+^ in the cells of insulin targeted tissues is associated with reduced activity of glucose transporter followed by the development of peripheral insulin resistance [110].

Intracellular Ca^2+^ concentration in insulin-responsive tissues, including adipose tissue and skeletal muscle, is regulated by several mechanisms. The first mechanism involves the action of PTH that increases intracellular Ca^2+^ concentration in insulin-responsive tissues, including adipose tissue and skeletal muscle [124,125,126], as well as reducing insulin-induced transport of glucose [127,128]. Therefore, both growing intracellular Ca^2+^ concentration and the decreasing number of GLUT-1 and GLUT-4 on the cell membranes evoked by PTH promotes insulin resistance observed as reduced glucose uptake [128,129]. There is evidence that vitamin D deficiency is associated with increased PTH levels coexisting with insulin resistance [130,131]. Wright et al. have shown that vitamin D reduced insulin resistance in skeletal muscle as a result of elevation of intracellular Ca^2+^ concentration and strengthening of GLUT-4 translocation to the membrane of muscle cells and glucose uptake [132].

It has also been observed that vitamin D might decrease insulin resistance indirectly via the renin-angiotensin-aldosterone system (RAAS). It is well known that the RAAS system inhibits insulin action in peripheral tissues and regulates cellular Ca^2+^ concentration in skeletal muscle cells [132,133,134]. Interestingly, the increased expression of renin and secretion of angiotensin II, as well as 1,25(OH)_2_D_3_-mediated inhibition of renin biosynthesis have been observed in VDR-null mice [135,136,137]. Therefore, it was shown that vitamin D improved insulin sensitivity via inhibition of RAAS [138].

To conclude, vitamin D alleviates the insulin resistance state via regulation of Ca^2+^ level and RAAS action in insulin targeted tissue, including skeletal muscle and adipose tissue.

### 5.3. Vitamin D-Mediated Improvement of Insulin Sensitivity Is Connected with Insulin Signaling

Accumulating evidence uncovers multiple potential mechanisms by which vitamin D deficiency can contribute to insulin resistance. It is generally accepted that abnormalities in the insulin signaling pathway are responsible for the development of insulin resistance that is characterized by reduced reaction of target cells to circulating insulin.

It has been found that vitamin D mediated increase in insulin sensitivity occurs via binding of calcitriol to VDR [139], induction of IRs expression [140], and the activation of peroxisome proliferator-activated receptor delta (PPAR-δ) [141]. The latter is a transcription factor engaged in the mobilization and metabolism of fatty acids in skeletal muscle and adipose tissue. What is more, activated PPAR-δ decreased FFAs-mediated insulin resistance in skeletal muscle. It was shown that 1,25(OH)_2_D_3_ activated PPAR-δ and improved insulin sensitivity. [141]. Manna et al. documented that vitamin D improved glucose metabolism as a result of upregulation of the SIRT1/IRS1/GLUT-4 signaling cascade and enhanced glucose uptake in high glucose-treated C2C12 myotubes [142].

The genomic pathway induced by 1,25(OH)_2_D_3_ in pancreatic β-cells, which express both VDR and CYP27B1, stimulates insulin synthesis and secretion since VDRE is present in the promoter region of the insulin gene [113,116,143]. Relevantly, studies performed on mice with the lack of functional VDR revealed that after glucose load, insulin synthesis and secretion were impaired [144]. Vitamin D-mediated improvement of insulin sensitivity is connected with insulin signaling. As a result of 1,25(OH)_2_D_3_ -mediated transcriptional activation of IR gene, the number of IRs on the surface of insulin responsive cells increases. Thus, upregulation of the IR gene ensures proper insulin signaling [140,145,146] and in this way calcitriol maintains insulin sensitivity [140,145,147]. It is suggested that vitamin D deficiency is involved in the onset of insulin resistance as a consequence of reduced expression of IR [2]. However, the results of vitamin D-mediated activation of IR expression in the liver are unambiguous. George et al. reported that vitamin D supplementation upregulated liver expression of IRs in streptozotocin-induced diabetic rats [148]. On the contrary, several studies failed to identify alterations in IR expression in the liver of mice fed with high-fat diet or low-fat diet [149], as well as in streptozotocin-induced diabetic rats after vitamin D supplementation [150].

To sum up, vitamin D alleviates insulin resistance via improvement of insulin signaling.

### 5.4. Vitamin D Possesses Indirect Antioxidant Properties

The pathogenic mechanism of insulin resistance is complex and has yet to be fully elucidated. Undoubtedly, the trigger factor in insulin resistance is adiposity, especially visceral, which is accompanied by chronic hyperglycemia, oxidative stress, and low grade chronic inflammation [80,151]. Additionally, a balance in the physiologic redox state is crucial for normal β-cells function, glucose homeostasis, and insulin sensitivity [152,153,154]. Oxidative stress is an imbalance between the production of reactive oxygen species (ROS) and the efficacy of antioxidant defense system. Endoplastic reticulum (ER) stress, hyperglycemia, dyslipidemia, lipid peroxides, and nitric oxide synthase, as well as advanced glycation end-products are involved in ROS overproduction in the insulin resistance diabetic state. It is well recognized that oxidative stress may activate several factors contributing to the development of insulin resistance [155,156]. Inoguchi et al. found that hyperglycemia and FFAs might activate ROS production via PKC-dependent stimulation of NADPH oxidase [157]. It was also observed that increased production of ROS is a key activator of insulin resistance [158,159]. Moreover, the association between the degree of insulin resistance and oxidative stress is suggested to induce cellular damage [156,160,161]. ROS have the ability to directly oxidize and damage cellular macromolecules, i.e., DNA, proteins, and lipids. Additionally, ROS may act as a signaling molecule that activates numerous cellular stress-sensitive pathways, i.e., NF-κB, JNK/SAPK, p38MAPK, and hexosamine involved in cellular damage and related pancreatic β-cells dysfunction, insulin resistance, and diabetes complication [162].

It is generally known that the hyperglycemic state is a causative factor responsible for the overproduction of ROS and reduced ATP formation that in turn exerts an effect on Ca^2+^ homeostasis leading to β-cell exhaustion and reduced resting insulin secretion. Furthermore, it is well recognized that the elevated formation of ROS increases the release of Ca^2+^ from ER via sensitization of the ryanodine receptors (RYRs) and inositol 1,4,5-trophosphate receptors (InsP_3_Rs). Reduced ATP level diminishes the capability of the Ca^2+^ pumps in ER and plasma membrane to press out Ca^2+^ from the cytoplasm outside of the cell. The effect may stimulate an increase of Ca^2+^ level in the pancreatic β-cells that triggers excessive insulin secretion leading to exhaustion of pancreatic β-cells [2]. Therefore, the elevated level of ROS strengthens Ca^2+^ signaling and may contribute to the onset of diabetes.

It was also proposed that oxidative stress coexisting with diabetes/chronic hyperglycemia is a result of increased FFAs levels that exert an effect on the mitochondria leading to increased ROS production (i.e., superoxide, hydrogen peroxide, hydroxyl radical ions) [163,164,165,166]. It was also suggested that vitamin D may regulate cellular bioenergetics in the mitochondria via VDR in the nucleus. This effect is related to the upregulation of numerous components involved in mitochondrial function, including mitochondrial respiration [167,168]. Additionally, VDR is capable of entering mitochondrion via permeability transition pores [169] and controls its functions, however this mechanism is still not fully understood [170]. It has also been found that vitamin D deficiency is connected with a decline in the mitochondrial respiration process. This effect is a consequence of the reduction of proteins and nuclear mRNA molecules engaged in this process [167,168]. Decreased respiration leads to a drop of mitochondrial bioenergetics related to alterations in oxidative phosphorylation, reduced ATP formation, and increased production of ROS [2]. Reduced expression of complex 1 of the electron transport chain contributes to the decrease of ATP production and ROS overproduction. In turn, increased ROS level reduces the activity of the insulin signaling pathways via lowering of GLUT-4 gene transcription, phosphorylation of IRS, disturbances in insulin signaling, and changes of mitochondrial activity [171,172,173]. These observations are supported by the results of a study showing that 1,25(OH)_2_D_3_/VDR signaling inhibits the process of differentiation of brown adipose cells and mitochondrial respiration [174]. Recently, Ricca et al. have demonstrated that VDR-mediated action of vitamin D may protect cells from overproduction of ROS and excessive respiration that leads to cell damage [175]. Vitamin D controls the balance of mitochondrial respiration via maintenance of mitochondrial respiratory chain activity [176] and the regulation of expression of uncoupling protein 1 (UCP1). UCP1 is localized on the inner membrane of mitochondria and is engaged in the regulation of thermogenesis [11]. The role of vitamin D in the maintenance of normal activity of mitochondria may explain at least partially the privileged relationship between diabetes and vitamin D deficiency.

Vitamin D has been shown to decrease ROS production in adipocytes [177] via the regulation of cellular antioxidants expression such as glucose-6-phosphate dehydrogenase (G6PD), glutathione peroxidase (Gpx), TR [178]. Interestingly, vitamin D together with Klotho and Nrf2 may control the expression of numerous antioxidants including catalase, Prx-2, Prx-3, SOD ½, GSH, TR, G6PD, TRX, Trxrd-1, Gpx. It has been documented that vitamin D decreases the expression of NADPH oxidase which is responsible for the production of ROS [179], while increasing the expression of SOD [178,180]. Furthermore, vitamin D elevated the production of glutathione (GSH), a major redox buffer through the upregulation of glutamate cysteine ligase, glutathione reductase, and G6PD [181,182,183]. To conclude, it seems that antioxidant properties of vitamin D are indirect and related to its genomic and non-genomic action.

### 5.5. Vitamin D Controls the Expression of Epigenetic Genes

A link has been proposed between epigenetic mechanism and numerous diseases, including obesity and T2DM [184]. DNA methylation was found to be increased in obese individuals and declared to be one of the risk factors in the development of diabetes [185]. It was observed that vitamin D maintains the expression of DNA demethylases genes as a result of its genomic mechanism of action. Namely 1,25(OH)_2_D_3_-VDR-RXR complex and its interaction with VDRE regulates the expression of vitamin D-dependent DNA demethylases (i.e., Jumonji domain-containing protein 1A and 3 (JMJD1A and JMJD3) and lysine-specific demethylase 1 and 2 (LSD1 and LSD2)). These enzymes perform demethylation of the promoter regions of numerous genes (i.e., ZEB1, ZEB2, SNAIL), and thereby prevent their hypermethylation [2,186].

### 5.6. Vitamin D Ensures Normal Function of Adipose Tissue

Wamberg et al. showed that VDR is expressed in the subcutaneous adipose tissue (SAT) and visceral adipose tissue (VAT). The VDR expression is higher in the VAT of obese as compared to lean individuals. However, no difference was observed in the expression of VDR in SAT between obese and lean individuals [187]. The expression of VDR was also reported in primary adipocytes from obese patients [188], suggesting a possible role of vitamin D in the processes of adipose tissue development and metabolism. The effect of vitamin D on adipogenesis, adipocyte apoptosis, lipolysis, lipogenesis, thermogenesis, and inflammation has been recognized [188,189,190,191,192,193].

#### 5.6.1. Vitamin D Regulates Lipid Metabolism in Adipose Tissue

It is widely known that adipocyte lipolysis is under hormonal regulation. Lipolytic hormones, including catecholamines, act via β-adrenergic receptors leading to increases in cAMP, which then activate cAMP-dependent PKA. Activated PKA, in turn, phosphorylates and activates enzymes of lipolysis [194]. Increased lipolysis coexisted with upregulated expression of fat oxidation markers such as CPT1α, PGC1α, PPARα, UCP1, SIRT-1, hormone-sensitive lipase (HSL), and lipoprotein lipase (LPL) [189]. It was demonstrated that 1,25(OH)_2_D_3_ inhibited adipocyte basal lipolysis in human adipocytes culture. Vitamin D increased the intracellular Ca^2+^ level [195] that caused a reduction of cAMP and a decrease in phosphorylation of HSL. In addition, Larrick et al. [196] and Chang et al. [189] have observed that calcitriol elevates the release of glycerol under basal and β-adrenergically stimulated murine 3T3-L1 adipocytes [189]. Summarizing, vitamin D exerts a suppressive effect on lipolysis.

It has been shown that the non-genomic mechanism of vitamin D action is involved in the regulation of adipocyte lipogenesis. Shi et al. reported that 1,25(OH)_2_D_3_ elevated the intracellular Ca^2+^ level and then the activity of fatty acid synthase (FSA). The observed effect was blocked by membrane antagonists and mimicked by membrane VDR agonists [195]. 1,25(OH)_2_D_3_-dependent upregulation of FAS might be mediated by VDR [188,197]. The increased mRNA of LPL, protein expression of lipogenic enzyme fatty acid-binding protein (FABP), and elevated accumulation of TAG was observed in response to 1,25(OH)_2_D_3_ of differentiated subcutaneous human adipocytes of female and male donors with 25.6–50.9 kg/m^2^ BMI range [190]. Moreover, the incubation of human adipocytes with 1,25(OH)_2_D_3_ evoked an increase in FAS protein level [195]. Interestingly, the increased expression of FAS in adipose tissue was not observed in Sprague-Dewley rats (16 days) after injection of 1,25(OH)_2_D_3_ [198]. Taken together, vitamin D may modulate the expression of lipogenic enzyme, however, further in vivo human studies are required to clarify these findings.

#### 5.6.2. Vitamin D May Control Adipogenesis

The participation of vitamin D in the process of adipogenesis has been explored. Along with the progress of adipocyte differentiation, VDR expression gradually decreases [199,200]. On the one hand, vitamin D was identified to promote adipogenesis in human and primary mouse preadipocytes [190]. On the other hand, however, the suppressive effect of vitamin D on adipogenesis in mouse preadipocyte and 3T3-L1 cell line [199] and the inhibition of brown adipocyte differentiation have also been shown. Moreover, it is suggested that a high dosage of calcitriol may inhibit the early stages of adipogenesis in 3T3-L1 cells [199,200]. Calcitriol suppressive effect on adipogenesis is exerted via its action on numerous targets decreasing the expression of C/EBPα and PPARγ, antagonizing the activity of PPARγ sequestrating RXR, as well as decreasing C/EBPβ mRNA and protein expression [200]. Blumberg et al. demonstrated that calcitriol stimulated the expression of eight twenty-one (ETO), a C/EBPβ corepressor. This molecule inhibits the action of C/EBPβ transcriptional activity that is required for adipogenesis [199].

Many signaling molecules, members of the WNT family, are released during preadipocyte differentiation. Physiologically, the WNT/β-catenin pathway exhibits decreased expression during adipogenesis and is responsible for maintaining the preadipocytes in an undifferentiated state [201]. Vitamin D was found to inhibit differentiation of adipocytes via MAPK [202] and Wnt/β-catenin signaling pathways [11,203]. It was also shown that calcitriol was involved in the regulation of the expression of the nuclear WNT10B and β-catenin, thereby suppressing PPARγ in a VDR-dependent manner leading to the inhibition of adipogenic differentiation in 3T3-L1 preadipocytes [199]. In addition, Cianferotti et al. revealed that 1,25(OH)_2_D_3_ diminished the level of secreted frizzled-related protein 2 (SFRP2) expression through VDR-dependent WNT signaling triggering to the suppression of mouse bone marrow stromal cells (BMSCs) differentiation [204]. Interestingly, calcitriol also demonstrated an inhibitory effect both on the mRNA expression and on phosphorylation of extracellular regulated kinase (ERK) resulting in the suppression of adipocyte differentiation [202]. Mesenchymal stem cells undergo differentiation towards adipocytes showing an accumulation of lipid droplets with coexisting increased expression of FABP4, PPARγ, and FASN. Calcitriol was found to strengthen the expression of the above differentiation markers and accumulation of lipids [188]. Mahajan et al. observed that incubation of porcine mesenchymal stem cells (MSCs) with vitamin D activated both their differentiation and proliferation as a consequence of increased mRNA of adipocyte-binding protein 2 (AP2), PPARγ, and LPL [205].

To conclude, vitamin D exerts an effect on the expression of genes engaged in adipogenesis. However, the results of studies focused on its influence on adipogenesis are inconsistent. Thus, further studies are required to further our understanding of the effect of vitamin D on adipogenesis.

#### 5.6.3. Vitamin D Is Engaged in Apoptosis of Adipocytes

It is well documented that Ca^2+^ is a key player in apoptosis and due to the vitamin D effect on the regulation of intracellular Ca^2+^ level, it is suggested that the vitamin is involved in this process [206,207,208]. Sergeev et al. have proposed a mechanism of intracellular Ca^2+^ regulation and apoptosis by calcitriol in obesity. This process was initiated by the vitamin D-dependent stimulation of Ca^2+^ influx from the extracellular space and mobilization of Ca^2+^ ER stores via the InsP_3_ receptor/Ca^2+^ release channel (InsP_3_R) and voltage-insensitive Ca^2+^ channels (VICC). Then, the activation of μ-calpain by the sustained cytosolic Ca^2+^ signal was followed by the induction of Ca^2+^/calpain-dependent caspase-12. These activated proteases seem to be sufficient for the execution of apoptosis [207]. By contrast, Sun et al. reported that calcitriol inhibited apoptosis and stimulated the expression of genes that promote the proliferation of human subcutaneous adipocytes [192]. They demonstrated that low doses of vitamin D suppressed, whereas high doses of calcitriol induced apoptosis of differentiated 3T3-L1 cells [209]. It was also suggested that low doses of vitamin D_3_ counteracted adipocyte apoptosis by elevating the ATP yield and mitochondrial potential, as well as suppressing UCP2 [210].

To summarize, decreased level of vitamin D in obesity and the action of 1,25(OH)_2_D_3_ to control mass of adipose tissue in vivo via regulation of adipocyte apoptosis may suggest a mechanistic role of vitamin D in adiposity [211]. However, further studies are required to better understand its role in adiposity and regulation of adipocyte apoptosis.

#### 5.6.4. Vitamin D Exerts an Effect on Thermogenesis

Thermogenesis is a process of heat generation and a key mechanism responsible for the maintenance of stable body temperature. In the management of obesity, thermogenesis reduces stored fat in β-oxidation process leading to the production of heat and ATP. Human studies have shown that vitamin D supplementation decreased body weight and fat mass [212,213]. Similarly, vitamin D may increase apoptosis of adipose cells in mice with diet-induced obesity which results in loss of weight [214]. Moreover, elevated fat oxidation and thermogenesis after vitamin D-rich breakfast intake show a direct relation between vitamin D and metabolism [215]. Brown adipose tissue expresses UCP1 that enhances thermogenesis. It has been shown that expression of UCP2 is inhibited by VDR after 1,25(OH)_2_D_3_ treatment of human adipocytes [193]. The increased expression of UCP2 driven by calcitriol leads to accelerated thermogenesis in mice with a high calcium diet. By contrast, Wong et al. have shown that VDR null mice have decreased storage of body fat, reduced level of cholesterol, and TAG under normal calcium concentration as compared to wild type mice suggesting that vitamin D did not mediate the loss of body fat. Additionally, white adipose tissue has shown a higher ratio of β-oxidation and upregulation of UCP1, UCP2, UCP3 in VDR-null mice as compared to wild type mice [216].

Taken together, the result of studies aimed to establish the effect of vitamin D on the regulation of thermogenesis and storage of body fat are inconclusive. Thus, further studies are required to better understand its role in these processes.

### 5.7. Vitamin D Decreases Low-Grade Chronic Inflammation Coexisting with Insulin Resistance

#### 5.7.1. Anti-Inflammatory Properties of Vitamin D Are Driven by the Reduction of Adipocytokine Production

It is well recognized that hypertrophic enlargement of adipose tissue is related to insufficient blood flow leading to hypoxia, macrophages infiltration, and inflammation, a typical feature of obesity [217,218]. The hypertrophied adipocytes overproduce pro-inflammatory cytokines such as TNF-α, IL-8, MCP1, IL-6, and resistin, as well as secreting less adiponectin [219,220,221]. In consequence, the dysregulation of many signaling pathways in hypertrophic adipose tissue causes insulin resistance [11,71,72,73,74,77,222,223,224]. Many acute phase proteins and pro-inflammatory cytokines are responsible for undesired crosstalk with biomolecules of insulin signaling [225]. For example, TNF-α and other pro-inflammatory cytokines influence the peripheral insulin sensitivity via the inhibition of insulin-dependent tyrosine phosphorylation of IRS-1. This effect then leads to improper activation of downstream insulin signaling molecules, including PI3-kinase and translocation of GLUT-4 to the cell surface [226,227]. It is known that angiotensin II and cytokines, i.e., monocyte chemotactic protein 1 (MCP1), TNF-α, IL-6, as well as other inflammatory factors engaged in inflammation may activate numerous intracellular protein kinases (i.e., JNK, S6 K, IKK, PKC) that phosphorylate IRS1 and IRS2 followed by attenuation of insulin signaling [133,219,226,228,229,230,231,232]. Therefore, low-grade chronic inflammation and increased adipose tissue infiltration of immune cells are strictly associated with both local and whole-body adipose tissue insulin resistance [229,233].

The action of vitamin D in adipose tissue is not only connected with the presence of VDR and enzymes involved in its metabolism, but also with its anti-inflammatory properties [11,234,235]. In vitro studies have shown that 1,25(OH)_2_D_3_ suppresses chronic inflammation in both mouse 3T3-LI cell line and human adipocytes as a result of diminished secretion of pro-inflammatory cytokines [233,236,237]. 1,25(OH)_2_D_3_ was also reported to decrease the activity of pro-inflammatory cytokines (i.e., MCP-1, IL-6, TNF-α, and IL-1β) in macrophages [229,233]. In turn, Chang et al. have shown increased protein and mRNA of TNF-α, IL-6, and macrophage infiltration in adipose tissue of Sprague-Dawley male mice fed with a high-fat low vitamin D content diet as compared to high-fat diet supplemented with 1000 IU of vitamin D per kg [238].

Another anti-inflammatory mechanism of vitamin D action found in murine adipocytes and human preadipocytes involves the inhibition of NF-κB/MAPK pathway [239,240,241,242,243]. NF-κB is known to be an important component of inflammatory pathways in adipose tissue. Its activation and translocation of p65 subunit to the nucleus is a result of IκBα degradation [244]. TNF-α- or LPS- stimulated receptors (i.e., IL-6R, TLR) activate NF-κB or p38MAPK-mediated transcription of pro-inflammatory genes (i.e., IL-6, IL-1β, and TNF-α). Cannel et al. showed that calcitriol inhibited IκBα phosphorylation and then translocation of NF-kB or p38MAPK into the nucleus [245]. Mutt et al. revealed that calcitriol suppressed the release of LPS-stimulated IL-6 in mature human adipocytes and differentiated MSC [240]. Moreover, it was demonstrated that 1,25(OH)_2_D_3_ reduced IL-1β-activated expression of pro-inflammatory genes including MCP-1, IL-6, and IL-8. However, the findings from in vitro studies are not in line with in vivo outcomes. On the one hand, Wamberg et al. reported that oral supplementation with vitamin D (700 IU per day) for 26 weeks did not reduce adipose tissue expression or circulating levels of IL-6, IL-8, and MCP-1 in obese patients [246]. On the other hand, in a mice model of obesity induced by a high-fat diet supplementation with calcitriol reduced IL-6 level in adipose tissue was observed [236].

It should be underlined that numerous cytokines activate kinases from both the Jun N-terminal kinase 1 (JNK1) and IKK-β/NF-κB pathways. In turn, these activated kinases phosphorylate IRS-1 leading to the impairment of insulin signaling [61,74]. Therefore, excessive secretion of pro-inflammatory cytokines triggers dysregulation of glucose and lipid metabolism [247]. Several studies demonstrated that vitamin D decreases monocyte chemotaxis, secretion of cytokines and chemokines playing an important role in reducing the extent of inflammation [70,76,80,81].

To conclude, numerous data indicate that vitamin D inhibits the release of pro-inflammatory cytokines [229,231,248], i.e., TNF-α, IL-6, and C-reactive protein [249,250]. The bioactive form of vitamin D strongly suppresses the activation of NF-κB and MAPK signaling pathways preventing transcription of pro-inflammatory genes. Thus, calcitriol significantly reduces the inflammation in adipose tissue.

#### 5.7.2. Vitamin D Regulates the Production of Adipokines

Recent data found that vitamin D was engaged in the regulation of adipokines release such as adiponectin and leptin [251,252,253]. Leptin released by adipose tissue acts on the hypothalamus that suppresses appetite and increases energy expenditure [254]. This hormone regulates the metabolism of lipids via the activation of lipolysis and suppression of lipogenesis [241,242]. Interestingly, the synthesis of leptin is induced by insulin, glucocorticosteroids, TNF-α, and estrogens, but it is suppressed by FFAs and growth hormones [255]. A positive correlation between leptin level and body fat mass is well documented [256]. It was revealed that vitamin D mediated, not only adipokines release, but also energetic homeostasis via the regulation of leptin formation. Another study showed that vitamin D suppressed the secretion of leptin by adipose tissue [257]. Additionally, CYP27B1 knockout mice were hypoleptinemic and consumed more food as compared to their wild type counterparts. In turn, VDR knockout (VDRKO) mice exhibited lean phenotype, hypoleptinemia, and hyperphagia related to reduced serum level of leptin [258]. The adipose tissue mass determines the level of leptin in the serum. It was not fully understood whether hypoleptinemia is a result of body fat content or is a direct result of action of the vitamin D/VDR system on leptin expression in VDRKO mice. 1,25(OH)_2_D_3_ directly initiates both the expression and secretion of leptin in wild-type mouse adipose tissue cultures. However, this effect was not observed in VDR-null mice adipose tissue cultures. It has also been found that 1,25(OH)_2_D_3_ decreases the expression of leptin by at least 84% in mouse 3T3-L1 adipocytes [259]. Notably, leptin was found to suppress the renal transformation of vitamin D_3_ to its active metabolite indirectly via the stimulation of osteoblast and/or osteocyte production of FGF-23 [260]. FGF-23 suppresses the synthesis of calcitriol via inhibition of renal CYP27B1 [261].

**Figure 5:**
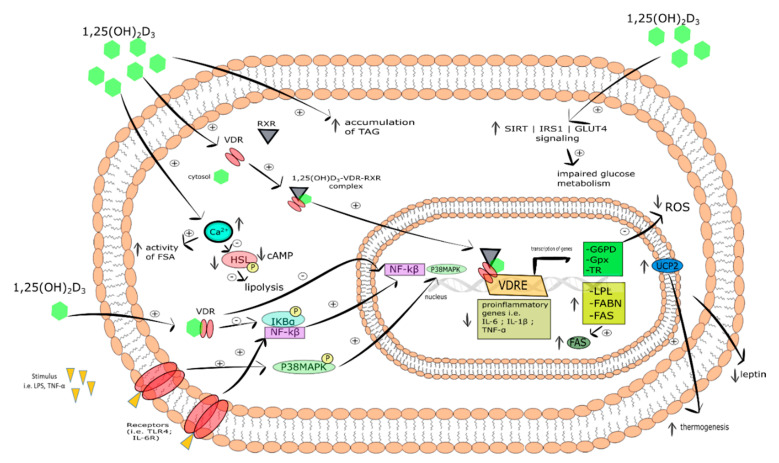
The effect of vitamin D on adipocyte function. Stimulatory interactions are indicated by + and inhibition by -; ↓ denotes decrease ↑ denotes increase.

Adiponectin is an anti-inflammatory and insulin-sensitizing hormone [262,263] that is considered as a biomarker of insulin resistance [255]. Its biological action depends on its serum level, the type of isoforms, and subtype of tissue specific receptor. The negative correlation between circulating adiponectin level and body mass index (BMI) is widely known. The downregulation of adiponectin, especially the HMW isoform has been found in obese children characterized by vitamin D deficiency [11,253]. Contrariwise, the elevated adiponectin level was identified in patients with T2DM supplemented with vitamin D-fortified food [264]. Moreover, calcitriol treatment increases adiponectin expression and disulfide bond-A oxidoreductase-like protein (DsbA-L). DsbA-L is a key protein involved in the multimerization of adiponectin [253]. On the one hand, no effect of calcitriol on the expression of adiponectin in human adipocyte culture was observed [265]. On the other hand, calcitriol was demonstrated not only to inhibit TNF-α –induced release of MCP-1, but also to suppress the release of adiponectin from differentiated adipocytes of subcutaneous VAT [266]. The effect of vitamin D on adipocyte function is summarized in Figure 5.

### 5.8. Vitamin D Regulates Lipid and Glucose Metabolism in Muscle Tissue and Liver

#### 5.8.1. Skeletal Muscle

Gilsanz et al. demonstrated that increased infiltration of fat in skeletal muscle tissue associated with insulin resistance and related vitamin D deficiency takes place independently of body mass increase [267]. Another study showed that the exposure of L6 and C2C12 myotubes to 1,25(OH)_2_D_3_ evoked increased expression of VDR, insulin receptor, and GLUT-4 [268,269]. What is more, it was demonstrated that the incubation of C2C12 myotubes with 1,25(OH)_2_D_3_ ameliorated lipid-induced insulin resistance by increased tyrosine phosphorylation of IRS-1 and serine phosphorylation of AKT [270,271]. Calcitriol was also able to increase mRNA of adipose triglyceride lipase (ATGL) and perilipin 2 (PLIN 2) in C2C12 myotubes, thereby revealing its engagement in intramuscular lipid catabolism and lipolysis [272].

Another mechanism of calcitriol action on skeletal muscle includes mitochondria. Ryan et al. found that the incubation of human primary muscle cells from healthy lean donors with calcitriol improved mitochondrial morphology and decreased mRNA of carnitine palmitoyltransferase 1 A (CPT1A) and pyruvate dehydrogenase kinase 4 (PDK4) which participate in muscle lipid and glucose metabolism [273].

**Figure 6:**
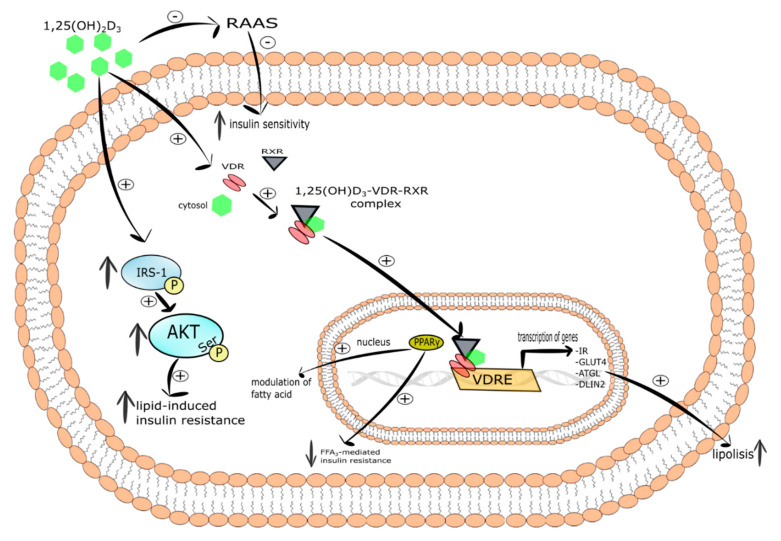
The mechanism of vitamin D action in myocyte. Stimulatory interactions are indicated by + and inhibition by -; ↓ denotes decrease ↑denotes increase.

Taken together, vitamin D may exert an effect on muscle insulin sensitivity by the improvement of glucose and lipid metabolism, mitochondrial function, and lipid turnover. However, further studies are needed to determine the importance of vitamin D in insulin sensitivity in muscle tissue. The mechanism of vitamin D action in myocyte is presented in Figure 6.

#### 5.8.2. Liver

It is well recognized that obesity is associated with excessive deposition of fat in the liver being a result of hepatic insulin resistance and inflammation [274]. Kong et al. have shown that intrahepatic injection of calcitriol in a dose of 5 ng/g body weight, twice per week for four weeks to vitamin D deficient mice fed with a high fat diet evoked the downexpression of pro-inflammatory markers and genes connected with hepatic lipogenesis, as well as the upregulation of bile acid transport [275]. Similarly, intraperitoneal injection of 1,25(OH)_2_D_3_ in a dose of 2.5 ng/g body weight, three times per week for four weeks decreased liver fat accumulation in mice [276].

Vitamin D was reported to affect hepatic lipogenesis and gluconeogenesis. This action is mediated via numerous vitamin D-regulated pathways, i.e., Akt/Notch signaling and/or AMP-activated protein kinase (AMPK)-calmodulin. The enzymatic property of AMPK is induced by phosphorylation through either serine/threonine kinase 11 pathways or the calcium/calmodulin protein kinase beta (CaMKKβ). Hepatic AMPK activation leads to the promotion of lipid oxidation and glycolysis, as well as attenuation of lipogenesis and gluconeogenesis [277]. Moreover, the induction of AMPK suppresses Foxo1 activity [278] causing the decrease in ER stress and attenuation of insulin resistance and steatosis [279,280]. It has been demonstrated that high doses of calcitriol ameliorates the abnormal hepatic glucose and lipid metabolism in insulin resistance models without toxicity symptoms. Lin et al. have shown that the elevated level of cytosolic 1,25(OH)_2_D_3_ in HepG2 cells evoked the induction of Ca^2+^/CaMKKβ/AMPK pathway. Additionally, calcitriol was found to ameliorate hepatic steatosis by the upregulation of autophagy-related mRNA genes such as ATG16L1 showing that vitamin D may stimulate lipophagy by the autophagy-lysosomal pathway [276]. Nelson et al. have shown an inverse correlation between the serum level of 25(OH)D_3_ and hepatocyte damage in obese women and men with steatohepatitis [281]. However, no correlation has been observed between the serum vitamin D level and fat accumulation in the liver or insulin sensitivity in obese women and men with steatohepatitis [282].

**Figure 7:**
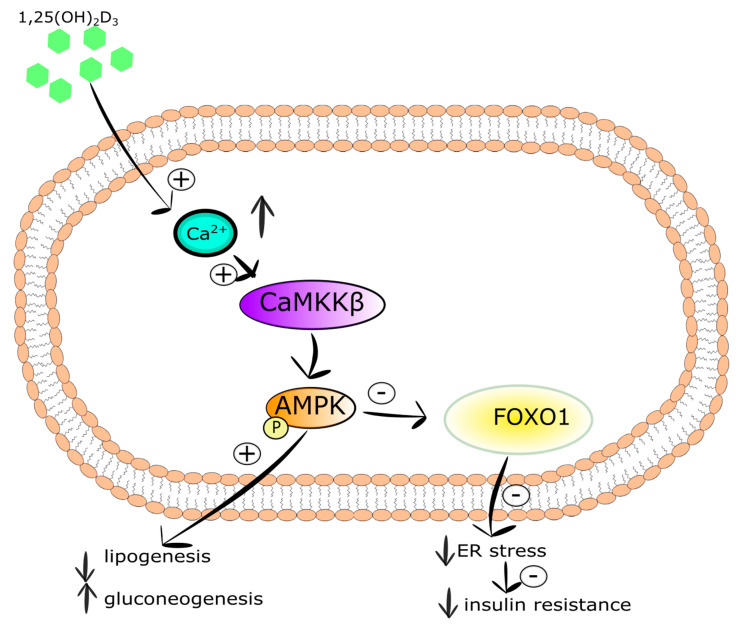
The action of vitamin D in hepatocyte. Stimulatory interactions are indicated by + and inhibition by -; ↓ denotes decrease ↑ denotes increase.

To summarize, vitamin D may ameliorate hepatic lipid and glucose abnormalities in vitro and in vivo via activation of Ca^2+^/CaMKKβ/AMPK signaling. The action of vitamin D in hepatocyte is shown in Figure 7.

### 5.9. Immunomodulatory Function of Vitamin D

The immunomodulatory function of calcitriol is widely known [29,283]. Vitamin D not only decreases adipose tissue inflammation by influencing leukocyte infiltration and maturation of adipocytes [284,285], but also acts on both the innate and adaptive immune system [245]. Vitamin D reduces the secretion of pro-inflammatory cytokines such as IFN-γ, TNF-α, IL-12 and increases production of anti-inflammatory cytokines (IL-10). Additionally, it was recognized that dendritic cells acquire an immunoregulatory function and tolerogenic properties as a result of vitamin D action [286]. It is also known that vitamin D reduces the expression and secretion of pro-inflammatory cytokines such as TNF-α, IL-1β, IL-8, and IL-6 in monocytes [287,288]. Moreover, vitamin D is involved in the change from more inflammatory response of T-helper 1 (Th1)/Th17 to less inflammatory Th2/Treg profile in lymphocytes [289]. Interestingly, the T lymphocyte activity is modulated in obesity [290]. Regulatory T cells (Treg) are subtypes of T lymphocytes. Treg are significantly decreased in VAT in obese mice [291]. Recent evidence has shown two crucial immunological mechanisms associated with insulin resistance namely, obesity-dependent and obesity-independent. Obesity-dependent insulin resistance is characterized by macrophage-driven inflammation [292]. In turn, obesity-independent mechanism involving age-related insulin resistance is controlled via adipose-resident regulatory T cell (aTreg) [293]. Calcitriol was demonstrated to reduce inflammation through strengthening the suppressive Tregs activity [291]. However, the action of vitamin D on aTreg is still not fully understood [294].

Considering the presence of numerous immune cells in adipose tissue and vitamin D actions, it alleviates the inflammation driven by insulin resistance [295].

## 6. Conclusions

Apart from mineral and bone metabolism regulation, vitamin D is also involved in a great number of cellular processes responsible for the homeostasis of glucose and lipid metabolism via insulin signaling pathway. Accumulating evidence supports that vitamin D deficiency is associated with the pathogenesis of insulin resistance. Disturbances in insulin signaling and inflammation are closely related, and vitamin D was found to reduce both of these disorders. Current evidence suggests that these benefits are the effect of vitamin D on Ca^2+^ and ROS homeostasis, as well as regulation of the expression of numerous genes. Considering multiple targets of vitamin D, we propose that pleiotropic action of vitamin D is a result of the crosstalk between insulin signaling and other signaling pathways governing metabolism, inflammation, immunomodulation, apoptosis, and adipogenesis. We have only just started to understand how vitamin D reduces insulin resistance and associated disorders. However, we would like to underline that although the awareness of vitamin D–associated health benefits is arising, the elevated consumption of vitamin D supplements may predispose to an increased incidence of vitamin D toxicity. Thus, without medical supervision, we advise caution for people who self-administrate higher than recommended doses of vitamin D.
